# Supplementary material for: Cardiolipin exposure on the outer mitochondrial membrane modulates α-synuclein
Source: Nat Commun. 2018 Feb 26;9:817. doi: 10.1038/s41467-018-03241-9 (PMC5827019; doi:10.1038/s41467-018-03241-9)
Supplement: Supplementary file 1 — Supplementary Information [file 41467_2018_3241_MOESM1_ESM.pdf]

Supplementary Information

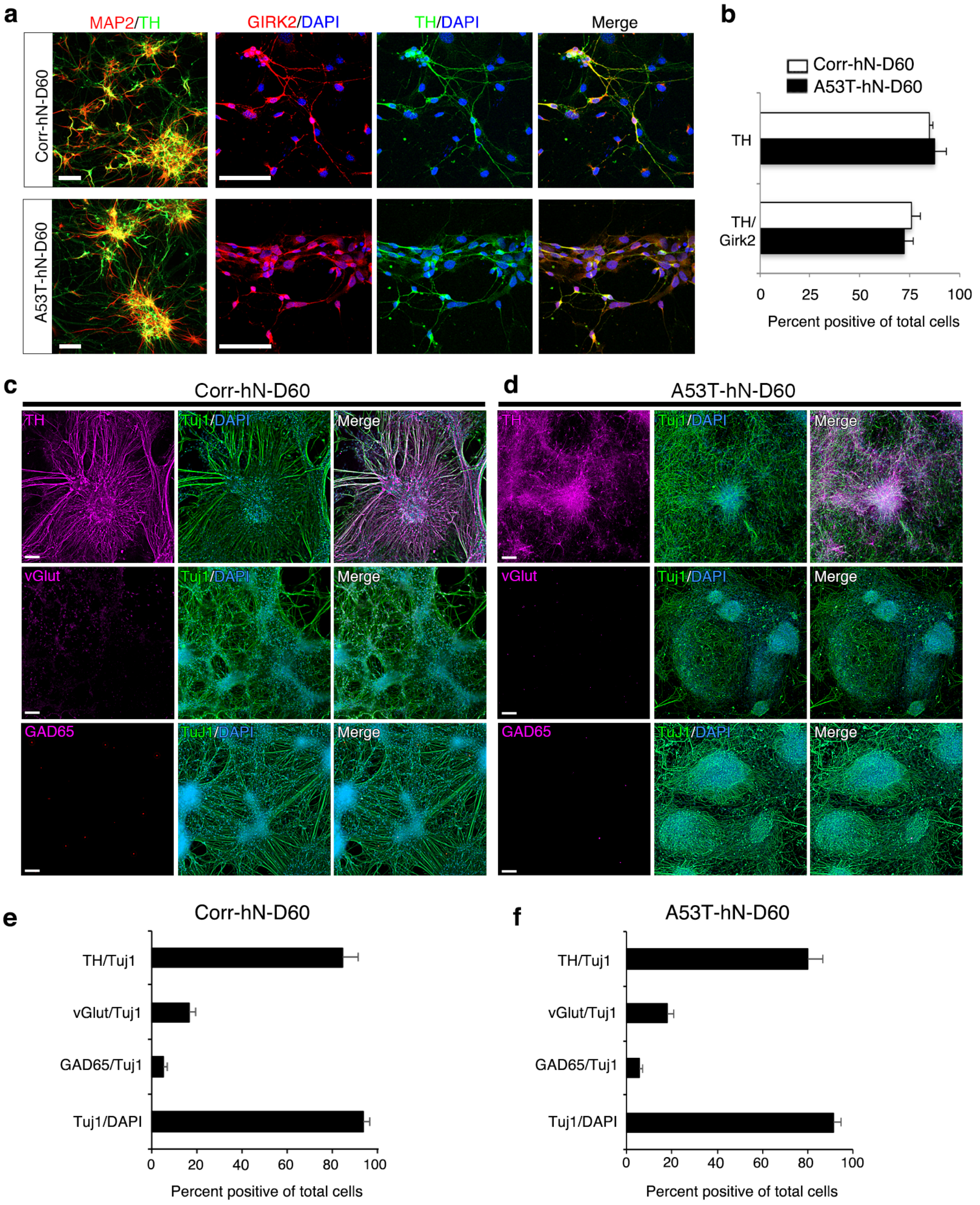

Supplementary Figure 1 | Characterization of floor plate differentiation. a-f, Quantification of neuronal subtypes present in differentiated hNs. hiPSC-derived corrected hNs (top panels) and A53T hNs (bottom panels) are both MAP2/TH double-positive and Girk2/TH double-positive at 60 days *in vitro* (DIV). Blue: DAPI. Scale bar: 50 µm (**a**). Quantification of TH-positive and TH-GIRK2 double positive populations. Data represent mean ± s.e.m., *n* = 6 coverslips over three independent differentiations, DIV: 60 (**b**). A53T and corrected hNs were antigenically labeled for Tuj1 and either vGlut, Gad65 or TH (**c,d**) and the percentage of cells expressing each marker was quantified (**e,f**). *n* = 6 coverslips from 2 independent differentiations, Blue=DAPI.

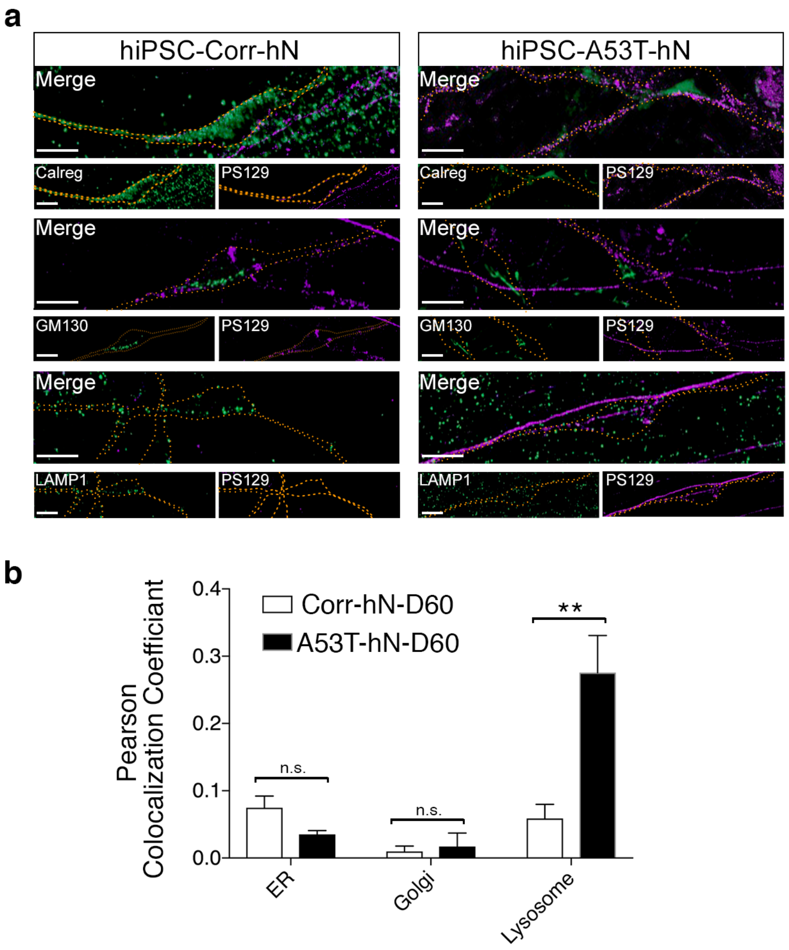

Supplementary Figure 2 | Subcellular localization of α-syn-PS129 in α-syn mutant hNs. a,b, Micrographs depicting α-syn-PS129 co-localization with the endoplasmic reticulum (Calregulin), the Golgi Apparatus (GM130) and the lysosomal compartment (LAMP1) (**a**). Quantification of Pearson co-localization coefficients from samples in panel a (**b**). Data represent mean + s.e.m. $^{**}P < 0.01$ by ANOVA, posthoc Tukey. $n = 4$ coverslips from 2 independent differentiations, DIV: 60.

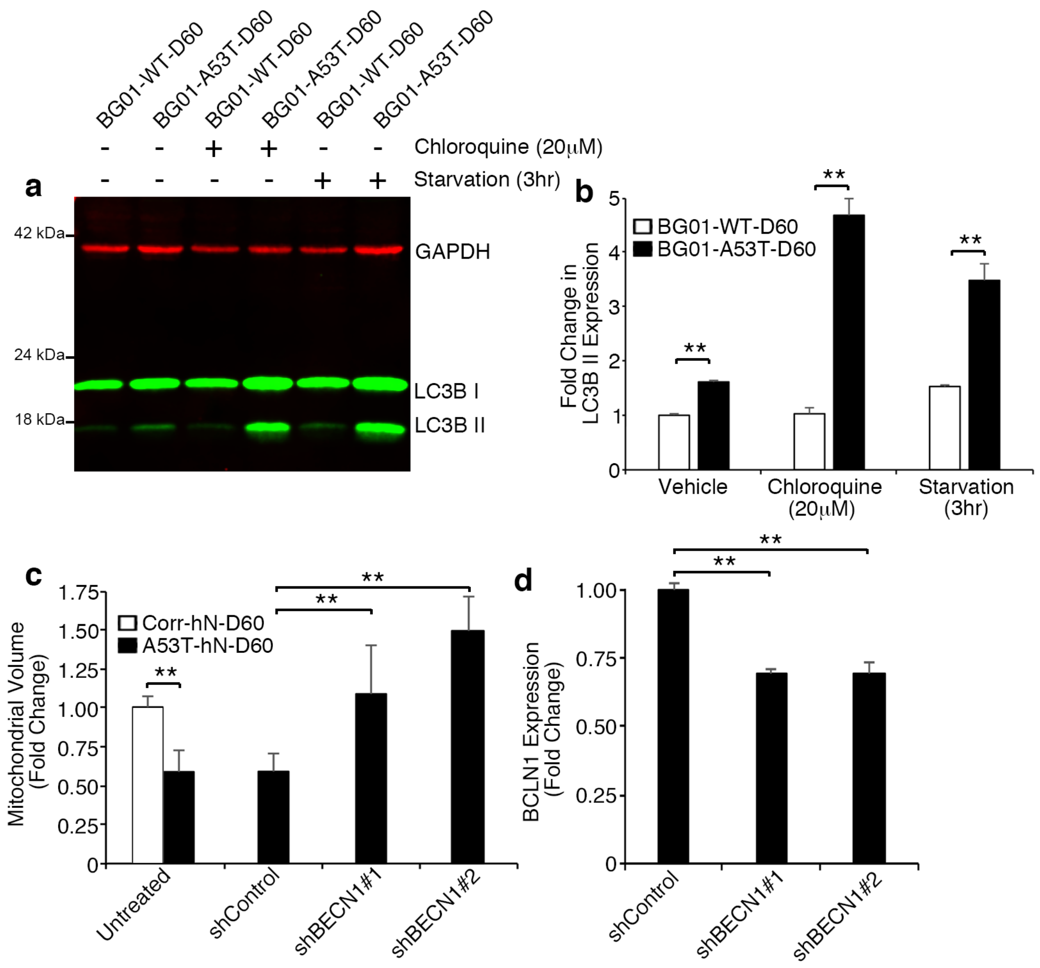

Supplementary Figure 3 | Characterization of mitochondrial pathology in BG01-WT and BG01-A53T hNs. a-b Steady-state levels of LC3I and LC3II protein expression were monitored by lysosomal inhibition with 20μM chloroquine (**a**). Turnover was induced with 3hrs of starvation. Triplicate experiments were quantified (**b**) relative to GAPDH expression. Data represent mean + s.e.m. $^{**}P < 0.01$ by Student t-test, $n = 3$. **c,** Quantification of the average mitochondrial volume per neurons for A53T and WT hNs following knock down of the mitophagy gene, Beclin-1 (*BECN1*) or scrambled shRNA control. Data represent mean + s.e.m. $^{**}P < 0.01$ by ANOVA followed by Dunnett, $n = 3$ neurons from 2 independent differentiations, DIV: 60. **d**, Knockdown efficiency of Beclin1 as measured by fold change in mRNA expression. Data represent mean + s.e.m. $^{**}P < 0.01$ by ANOVA followed by Dunnett, $n = 3$ independent experiments.

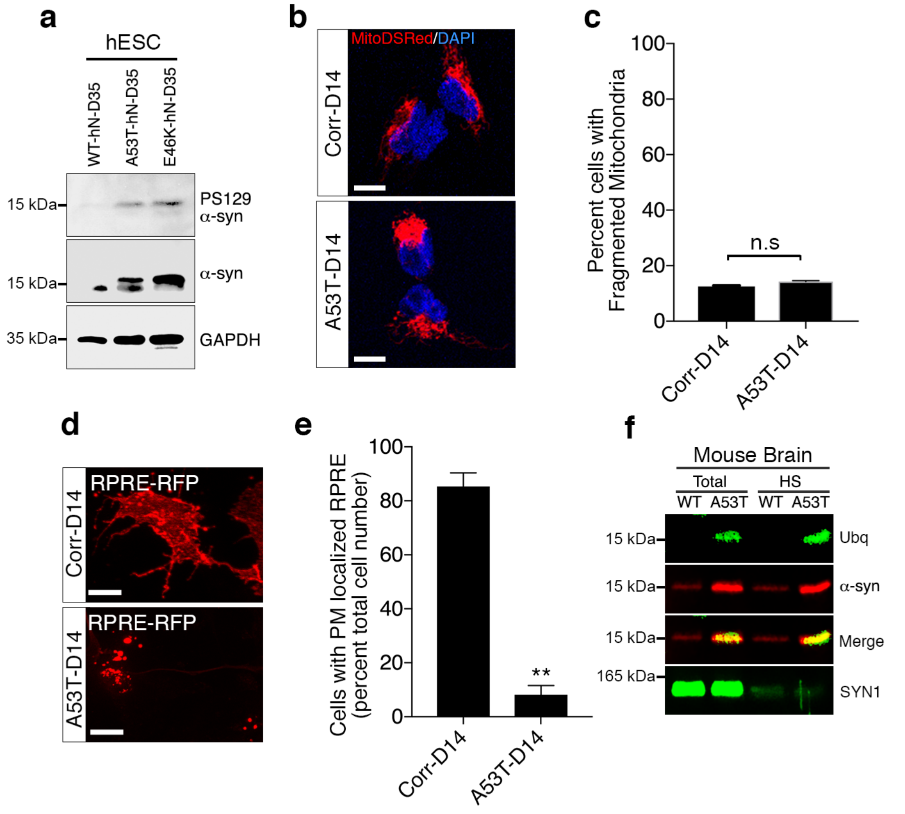

Supplementary Figure 4 | Characterization of α-syn accumulation and mitochondrial fragmentation. a, Western blot analysis of lysates from hESC-derived WT, A53T or E46K hNs at DIV 35 labeled for total α-syn, PS129, or GAPDH shows that levels of PS129 modified α-syn (14 kDa) are elevated in A53T and E46K hNs. **b,c,** Mitochondrial structure is normal in in hiPSC-derived A53T cells at the NPC stage (DIV14) as shown by labelling with MitoDSRed (**b**). Quantification of data (**c**) represent mean ± s.e.m. $P = 0.1647$ by Student *t*-test, $n = 8$ coverslips over 2 independent differentiations, DIV: 14; scale bar: 10 μm. **d,e,** Micrographs of hiPSC-derived corrected (left panel) and A53T mutant NPCs at DIV 14, expressing the negative charge probe RPRE-RFP (**d**). RPRE shifts from a primarily plasma membrane localization in isogenic-corrected hNs to a punctate intracellular localization in A53T hNs at DIV 14. Quantification of percentage of hNs with plasma membrane localized RPRE-RFP at DIV14 (**e**). Data represent mean ± s.e.m. $^{**}P < 0.0001$ by Student *t*-test, $n = 6$ coverslips over 2 independent differentiations, DIV: 14; scale bar: 10 μm. **f,** Western blot analysis of total and heat stable (HS) lysate from WT and A53T transgenic brain probed for ubiquitylation (Ubq), α-syn or synaptophysin 1. Merge represents overlay of α-syn and Ubq.

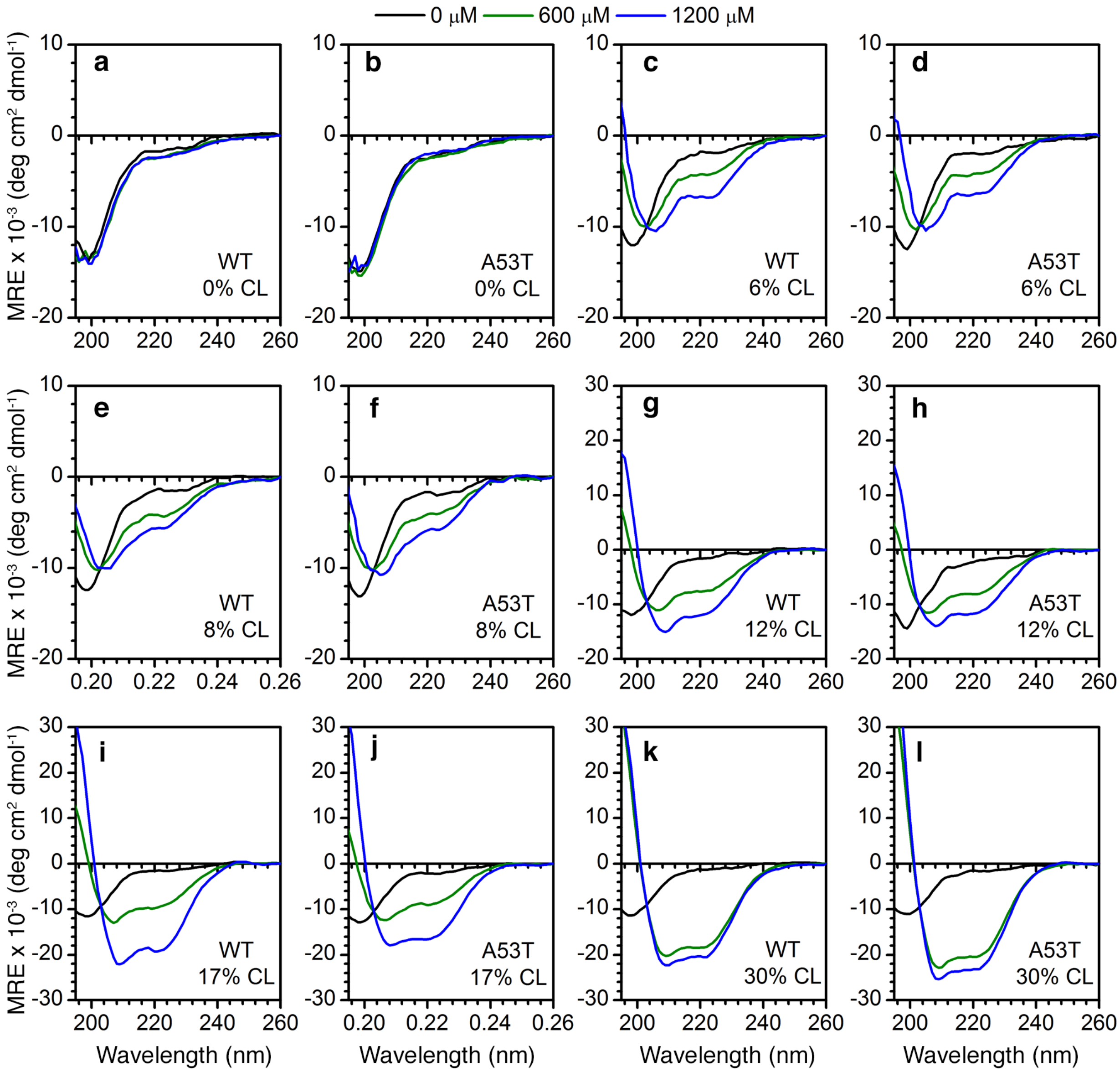

Supplementary Figure 5 | Effect of large unilamellar lipid vesicles (LUVs) with different cardiolipin (CL) content on folding of WT and A53T α-syn monomers. a-l, Six lipid compositions of PI:PC:PS:PE:CL with increasing molar ratio of cardiolipin were used, namely, 9.4:55.2:7.3:28.1:0 (**a**,**b**); 8.8:51.9:6.9:26.4:6 (**c**,**d**); 8.6:50.8:6.7:25.9:8 (**e**,**f**); 8.3:48.6:6.4:24.7:12 (**g**,**h**); 7.8:45.8:6.1:23.3:17 (**i**,**j**); and 6.6:38.6:5.1:19.7:30 (**k**,**l**). Monomeric WT (**a**,**c**,**e**,**g**,**i**,**k**) or A53T (**b**,**d**,**f**,**h**,**j**,**l**) α-syn (4 μM) was incubated in the presence of an increasing concentration of LUVs at 37°C, and the CD spectrum was collected to follow the relative redistribution of the secondary structure components (from mostly random coil in the absence of LUVs to α-helical structures in their presence).

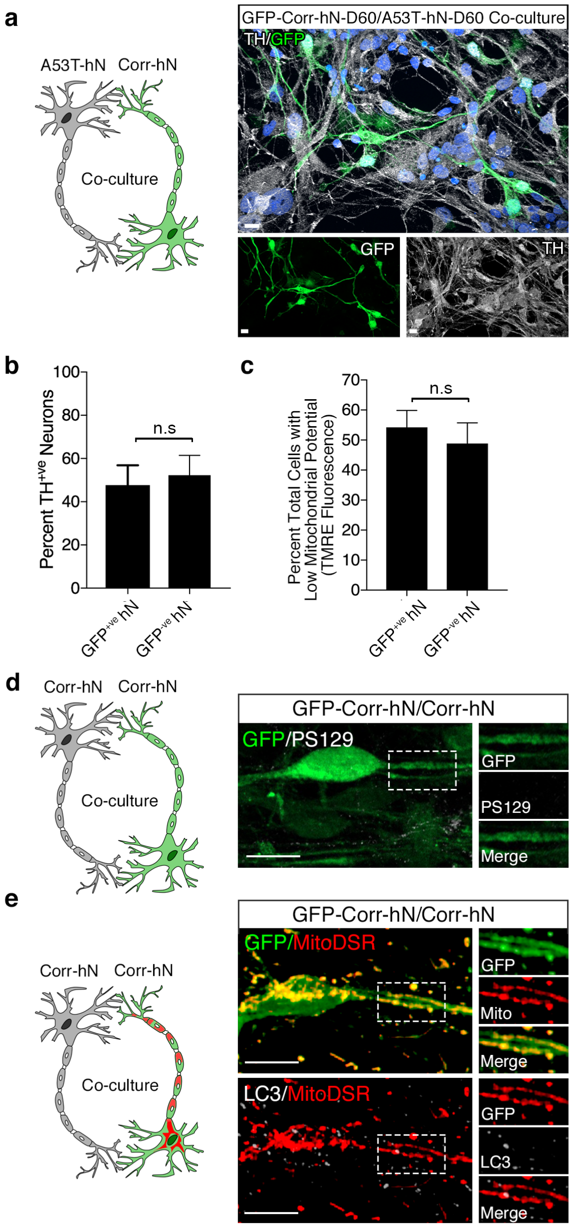

Supplementary Figure 6 | Characterization of differentiation of A53T and isogenic-corrected hNs in co-culture. a,b, Micrographs of GFP^{+ve} isogenic-corrected hNs and GFP^{-ve} A53T mutant hNs in co-culture, antigenically-labeled for TH (**a**). Percentage of total TH positive neurons that are GFP^{+ve} and GFP^{-ve} (**b**). Data represent mean + s.e.m. *P* = 0.3022 by Student *t*-test, *n* = 10 coverslips over 2 independent differentiations, DIV: 60. **c**, Percentage of total neurons with depolarized mitochondria. Data represent mean + s.e.m. *P* = 0.5764 by Student *t*-test, *n* = 3 cultures from independent differentiations, DIV: 60.**d,** GFP^{+ve} isogenic-corrected hNs and GFP^{-ve} isogenic-corrected hNs in co-culture, antigenically-labeled for PS129 modified α-syn. **e,** GFP^{+ve}, MitoDSRed^{+ve} isogenic-corrected hNs and GFP^{-ve} isogenic-corrected hNs in co-culture, antigenically-labeled for LC3. Scale Bar: 10 μm.

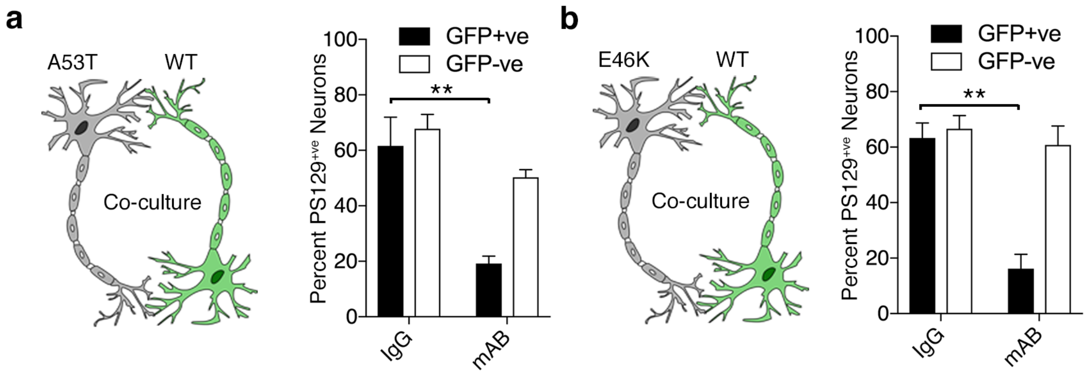

Supplementary Figure 7 | Blocking α-syn transmission blocks α-syn PS129 accumulation. a-b, hESC-derived WT cells expressing GFP were co-cultured with either A53T cells (**a**) or E46K cells (**b**) at DIV 14 and differentiated to hNs (DIV 60) together in the presence of either monoclonal anti-α-syn or IgG control to block α-syn seeding and percentage PS129 positive neurons was quantified. Data represent mean + s.e.m. $^{**}P < 0.0001$ by ANOVA, posthoc Tukey, $n = 5$ coverslips from 3 independent differentiations, DIV: 60.

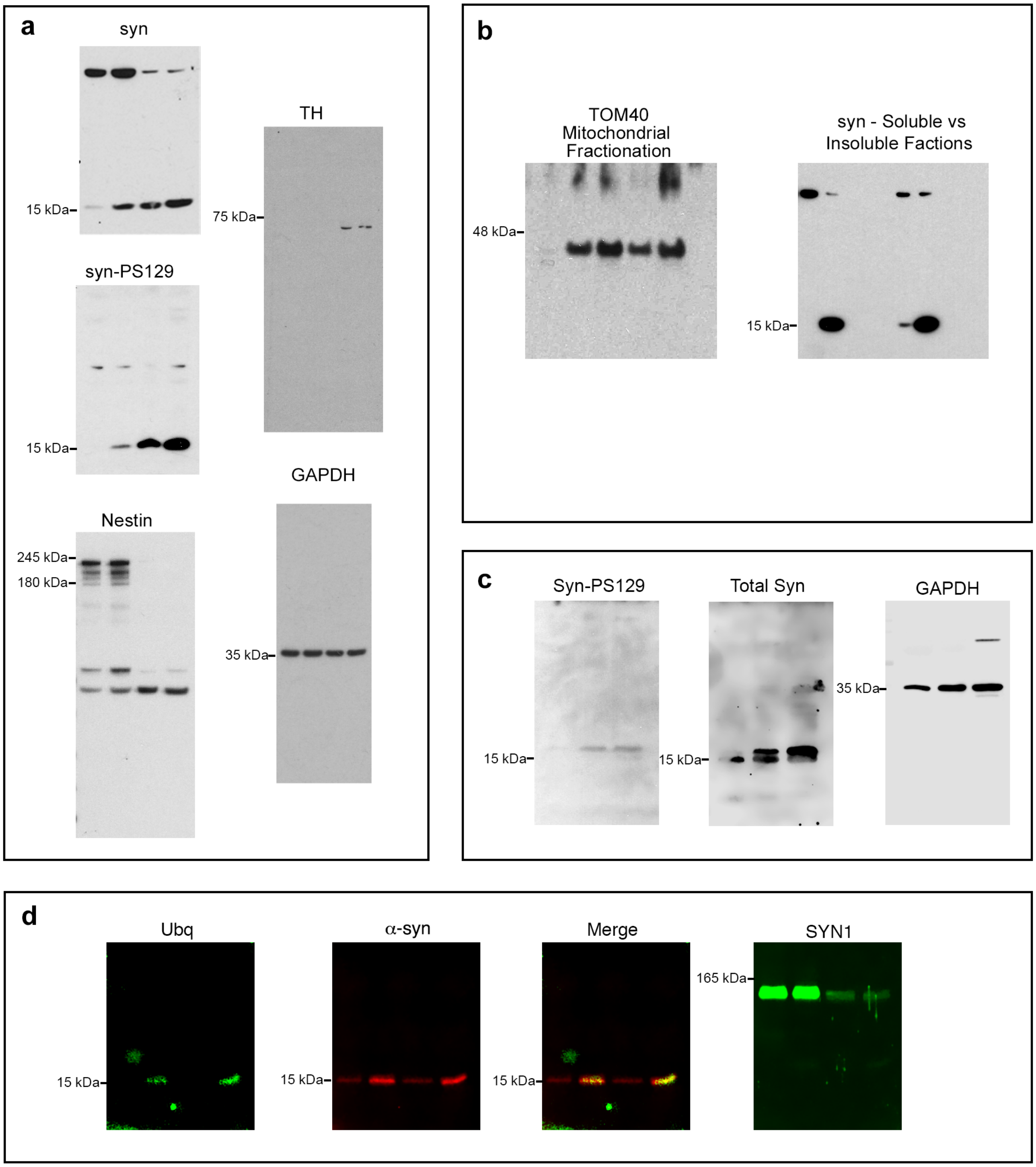

Supplementary Figure 8 | Uncropped blots. a-d, Uncropped blots from figure 4a (**a**), figure 4b (**b**), and supplementary figures S4a (**c**) and S4f (**d**) are depicted.

Supplementary Table 1: Binding parameters of WT, A53T and E46K α-syn to cardiolipin-containing LUVs

Type of LUV	WT				A53T				E46K			
	K_D, μM	n	L	R^2	K_D, μM	n	L	R^2	K_D, μM	n	L	R^2
8% CL	~ 3.7 mM	N.D.	N.D.	0.946	~ 4.2 mM	N.D.	N.D.	0.961	140.74 ±27.14	166.27 ±19.77	13.30 ±1.58	0.994
12% CL	199.69 ±22.46	296.00 ±16.22	35.52 ±1.95	0.998	438.11 ±54.61	202.00 ±32.24	24.24 ±3.87	0.996	56.59 ±12.74	146.77 ±11.44	17.61 ±1.37	0.996
17% CL	52.02 ±11.62	220.98 ±11.04	29.57 ±2.31	0.997	114.44 ±24.68	163.91 ±18.11	27.87 ±3.08	0.995	11.29 ±6.18	130.85 ±3.72	22.25 ±1.32	0.995
30% CL	1.11 ±2.33	98.51 ±7.71	29.57 ±2.31	0.993	2.36 ±4.13	95.34 ±10.31	28.6 ±3.09	0.987	8.37 ±4.69	86.34 ±6.21	25.90 ±1.86	0.994

Binding parameters (K_D, n, and L) were obtained from fitting of the data presented in Figure 5 (a-c) to equation 1 (see Methods). s.e.m was estimated by the fitting program (OriginPro 8) at the confidence level of 95%. n – the total number of lipid molecules in the bilayer that are involved in binding one molecule of protein (stoichiometry of binding). L – the number of cardiolipin (CL) molecules in the bilayer that are involved in binding one molecule of protein. Date represent mean ± s.e.m. of 3 replicates.

Supplementary Table 2: Efficacy of mAB-α-syn mediated capture of α-syn from conditioned media

	Corr-hN-D60	A53T-hN-D60	Co-Culture
**α-synuclein captured (ng ml^{-1})**	10.85±4.75	6.05±2.80	6.98±1.43

One mL of conditioned media from hiPSC-derived A53T, Corrected or Co-Cultured hNs was incubated for 24 hours at 37°C with 1ug of α-syn-mAB conjugated to protein G dynabeads. The quantity of captured α-syn was normalized against a standard curve of recombinant human α-syn. Date represent mean ± s.d. of 4 replicates. Mean values do not differ significantly.
